# Highlights from the 9th Cachexia Conference

**DOI:** 10.1002/jcsm.12217

**Published:** 2017-06-20

**Authors:** Nicole Ebner, Stephan von Haehling

**Affiliations:** ^1^ Department of Cardiology and PneumologyUniversity Medical Center GöttingenGöttingenGermany

**Keywords:** Cachexia, Muscle wasting, Sarcopenia

## Abstract

This article highlights updates of pathways as well as pre‐clinical and clinical studies into the field of wasting disorders that were presented at the 9th Cachexia Conference held in Berlin, Germany, December 2016. This year, some interesting results from clinical trials and different new therapeutic targets were shown. This article presents the biological and clinical significance of different markers and new diagnostic tools and cut‐offs of detecting skeletal muscle wasting. Effective treatments of cachexia and wasting disorders are urgently needed in order to improve the patients' quality of life and their survival.

## Introduction

The development of preventive and therapeutic strategies against cachexia and wasting disorders, such sarcopenia, is perceived as an urgent need by healthcare professionals and has instigated intensive research into the pathophysiology of these syndromes.^1^, ^2^ Cachexia is characterized by progressive weight loss affecting different body compartments, particularly skeletal muscle and adipose tissue, although even bone mineral content may be affected.^3^ Over the last years, the Cachexia Conference has developed into a respected forum for researchers from the fields of cachexia and wasting disorders. It is unique in several ways as it provides a platform for both clinicians and basic researchers to meet and discuss pathways and potential therapeutic targets as well as recent evidence from clinical trials. The 9th Cachexia Conference was held in Berlin, Germany, from 10 to 11 December 2016 with over 250 participants from more than 25 countries attending.

## Basic science

This year, some interesting updates on signalling pathways were presented. One of these important pathways is the ubiquitin–proteasome system. The ubiquitin proteasome system plays a critical role in skeletal muscle wasting. Studies from many groups over the past years have indeed identified many components in the ubiquitin‐conjugating system that are induced in atrophying skeletal muscle. This year, Taillandier *et al.* (Unité de Nutrition Humaine, Clermont‐Ferrand, France) focused on E2 enzymes that are either abundant or up‐regulated in skeletal muscle wasting. They showed that the ubiquitin enzyme UBE2B is involved in contractile protein targeting. The autophagy family members include more than 30 components recruited to form the autophagosome. Among these, Atg8, which is soluble in the cytoplasm, is lipidated and cocked to the internal membrane. Therefore, the Atg protein is present during autophagy and is considered as a good marker to monitor the activity of the autophagy process.^4^ In this context, Kneppers *et al*. (Maastricht University Medical Center, Maastricht, The Netherlands) showed that parallel activation of autophagy and myogenic signalling exist during muscle mass recovery following disuse atrophy. The parallel changes in expression of autophagy and myogenic differentiation markers during early muscle reloading imply a co‐ordinated regulation of these processes during recovery of muscle mass following atrophy.^5^ Musolinio *et al*. (University of Catanzaro, Rome, Italy) showed musculoskeletal changes associated with chronic constriction injury of the rat sciatic nerve. Interestingly, the modulation of anabolic and catabolic pathways, focusing on autophagy, in skeletal muscle, using a model of atrophy induced by chronic sciatic nerve constriction showed that muscle atrophy induced by chronic sciatic nerve constriction might have different mechanisms than common muscle atrophic models.^6^ At 14 days post‐sciatic nerve ligation, the chronic constriction injury group had significant decreases in body weight and body composition compared with naive animals. The ubiquitin E3‐ligases atrogin‐1 was decreased, suggesting that its involvement could occur at an earlier time point. Also, levels of p‐Akt and total Akt were up‐regulated, a result expected in a hypertrophic model. Guttridge *et al*. gives an overview to the important functions of transcription factors nuclear factor *κ*B (NF‐*κ*B) in myoblast to stimulate activin and inhibit muscle differentiation. He impressively showed that NF‐*κ*B is activated in cachectic muscle in both PAX 7 progenitor cells and myofibres.^7^ Other studies buttress the view that increased FoxO signalling and the activation of the transcription factors NF‐*κ*B, muscle RING‐finger protein (MuRF1), and muscle atrophy F‐box (MAFbx) in skeletal muscle play major roles during cachexia onset and progression.^8^, ^9^, ^10^ MuRF1 and MAFbx are essentially involved in muscle atrophy development. Indeed, genes whose expression levels are commonly increased during multiple models of skeletal muscle atrophy, including cancer and sepsis, are MAFbx, MuRF1, and cathepsin, and there is evidence that each is FoxO target genes. Inducers of MuRF1 and MAFbx expressions are TNF*α*, IL‐6, and IL‐1, and NF‐*κ*B appears to be the most important regulator of MuRF1 and MAFbx expressions in the skeletal muscle.^11^ Another new target for therapeutic interventions seems to be TAK1, which is required for the growth and maintenance of skeletal muscle mass. Kumar *et al.* (University of Louisville School of Medicine) showed that the inactivation of TAK1 in skeletal muscle causes loss of body mass and strength. Moreover, they impressively show that the inactivation of TAK1 stimulates slow‐to‐fast‐type myofibre transition in soleus muscle and reduces the rate of protein synthesis in skeletal muscle in mice. They conclude that TAK promotes protein synthesis potentially through activation of mTOR. The loss of TAK1 causes oxidative stress and activates proteolytic pathways in skeletal muscle, and the inactivation of TAK1 leads to the activation of AMPK and Smad2/3 in skeletal muscle. TAK is essential for the overload‐induced skeletal muscle hypertrophy. A number of elegant models were presented in order to improve our understanding of pathways involved in the wasting process. Muscle wasting has received increasing research efforts in recent years.

## Body composition

During the congress, several different techniques were presented to measure body composition. These included computed tomography scan, dual energy X‐ray analysis and magnetic resonance imaging (MRI), D3‐creatine dilution analysis, and bioimpedance analysis (BIA). Most surprising data in the field of daily activity measurements presented Rantanen *et al.* (University of Jyväskylä, Finnland) with simple grip strength measurements. The force produced by the hand to press or squeeze correlates well with the results of other muscle strength tests, so it can be viewed as an easily applicable test of total body strength. The authors concluded that grip strength measurement can be used as a marker of physiological reserve during ageing. Grip strength declines by approximately 1–2% annually after midlife. Thus, it can be used as biomarker of ageing. Grip strength reflects the combined influences of genetic predisposition acquired modifications of physical constitution ageing processes chronic diseases. In this regard, Flores *et al.* (Universidad de Colima, Colima, Mexico) discussed the correlation between handgrip strength and muscle mass in 155 patients undergoing maintenance hemodialysis.^12^ Also Szulc *et al.* (University of Lyon, France) presented data of grip strength measured in the STRAMBO study. They showed that grip strength is associated with hormonal deficits and this remains true regardless of other confounders.^13^ The STRAMBO cohort consists of 811 men aged 60 to 87 years and grip strength was measured at baseline and after 4 and 8 years. The authors show impressively that increased high sensitivity C‐reactive protein levels and poor physical performance are associated with accelerated grip strength decline.

However the most amazing method was descripted by Stimpson *et al.,* 14 the D3‐creatine dilution for determination of total body creatine pool size and skeletal muscle mass. This interesting method can directly assess skeletal muscle mass or its change, during ageing, inactivity, disease, or exercise. This method takes advantage of a number of aspects of creatine biology. More than ninety percent of the total body creatine pool is found in skeletal muscle. Newly synthesized creatine from hepatic and renal sources is transported into the sarcoplasm against a large concentration gradient. ^2^H labelled creatine is ingested as a 30 mg capsule digested and distributed to skeletal muscle. Creatine is converted to creatinine and excreted in urine. Last year, Evans *et al.* (University of California, USA) presented results of a clinical validation study demonstrating that the creatine dilution method is strongly associated with whole body MRI‐method.^15^ This year they presented that the creatine dilution method is also strongly associated with the dual energy X‐ray analysis method. D3 creatine dilution method appears to be a valid non‐invasive measurement for muscle mass and children. In some adults, a portion of the ingested D3 creatine label is filtered by the kidney and spilled into urine. By measuring creatine/creatinine ratio, a correction for spilled label is determined with an algorithm. Combined with dosing of ^2^H_2_O dosing, lean body mass and muscle mass can be measured non‐invasively. Importantly, the measurement with creatine dilution method is not affected by shifts in body water that occur with many cachectic diseases.

## Clinical trials and newly treatment targets

Li *et al.* 16 (Acceleron Pharma, Cambridge, Massachusetts, USA) presented data of ACE‐2494, a new growth differentiation factor (GDF) ligand trap. They used an immobilization mouse model to specifically induce tibialis anterior muscle atrophy and tibiae bone loss. A total of 30 mice were immobilized for 14 days and receive ACE‐2494 (10 mg/kg) twice per week or vehicle. The results showed that ACE‐2494 treatment resulted in enlarged muscle fibre size by over 28% and restored the loss in area bone mineral density. Thus, ACE‐2494 restored and prevented muscle loss and can be used as a potential treatment for muscle atrophy and muscle disorders.^16^ Ábrigo *et al.* (Universidad Andres Bello, Chile, Santiago de Chile) showed that administration of angiotensin 1–7 as complex of hydroxyl polyamidoamine (PAMAM‐OH) avoided the atrophic effects in skeletal muscle atrophy.^17^ Mice were immobilized and treated with angiotensin alone and in combination with PAMAM‐OH or vehicle. They showed that only the combination of angiotensin with PAMAM‐OH dendrimer can be an efficient method in therapy for treatment of skeletal muscle atrophy. Also, Rivera *et al.* (Universidad Andres Bello, Chile, Santiago de Chile) showed that angiotensin 1–7 decreased Lipopolysaccharide (LPS)‐induced LC3II/LC3I ratio and suggested that angiotensin 1–7 is a new regulator of autophagy by mechanism dependent on MAPK.^18^ Interesting data in the field of the metabolic effects of a vitamin D and leucine‐enriched breakfast were presented by Paddon‐Jones *et al*.^19^ They showed that breakfast normally has the inadequate protein distribution that is why the study was designed to investigate the acute effect of supplementing breakfast with vitamin D and leucine enriched for 6 weeks with protein medical nutrition drink versus control group. Interestingly, appendicular leg lean mass was increased after 6 weeks compare with the control group. Additionally, the post‐prandial glucose peak was lower and delayed when breakfast was supplemented with vitamin D and leucine. On the basis of this work, the authors propose a novel and specific dietary approach to prevent or slow down muscle loss with ageing. To maximize muscle protein synthesis, they propose a dietary plan that includes 25–30 g of high‐quality protein per meal. In this regard, Laviano *et al.* (Sapienza Universita di Roma, Roma, Italy) presented data of a randomized controlled study investigating the effects of targeted medical nutrition (Remune, Smartfish) versus an isocaloric comparator in patients with chronic obstructive pulmonary disease (COPD).^20^ Remune is high in omega‐3 fatty acids eicosapentaenoic acid (EPA)/docosahexaenoic acid (DHA) and vitamin D and is a source of protein. The product is suitable for patients with pre‐cachexia and cachexia who are not able to get sufficient amounts of these nutrients through their normal diet, such as patients with COPD or cancer. Remune is a milk‐based drink and includes 226 kcal, 10 g protein, omega 3, and 10 μg vitamin D. A total of 45 patients with moderate to severe COPD with involuntary weight loss receive either targeted medical nutrition (Remune, Smartfish) or isocaloric comparator twice daily for 12 weeks. A significant increase in plasma vitamin D and omega 3 levels was shown. Only in the remune‐treated group a significant increase in fat mass and improvement in exercise capacity measured as 6 min walk distance test were shown.

Interesting results of phase II studies were this year presented. Karaa *et al.* (Massachusetts General Hospital, Boston, Massachusetts, USA) showed the effect of elamipretide previously known as MTP‐131 or bendavia in patients with primary mitochondrial disease.^21^ Patients (*n* = 36) with genetically confirmed primary mitochondrial disease and symptoms of exercise intolerance and fatigue were enrolled in a randomized, double‐blind, placebo‐controlled, multiple‐ascending dose study. This study tested the safety and efficacy of intravenous elamipretide in doses of 0.01, 0.10, and 0.25 mg/kg/h infused for 2 h once daily for 5 days. Treatment with elamipretide after 5 days resulted in a dose‐dependent increase in distance walked on the six minute walk test (6MWT) with few adverse events, which did not differ from those seen in controls. This first dose‐finding study suggests that elamipretide may reduce the extent of exercise disability in patients with primary mitochondrial disease.

Coats *et al.* 22 (Melbourne, Australia) presented the results of the ACT‐ONE trial with 87 patients from 17 centres. The ACT‐ONE trial, a multicentre, randomized, double‐blind, placebo‐controlled, dose‐finding study of the anabolic‐transforming/catabolic‐transforming agent espindolol recruited subjects with cachexia and non‐small‐cell lung carcinoma (NSCLC) or colorectal cancer in stages III and IV.^22^ Patients were randomized in a 3:1:2 fashion to one of two doses of espindolol (10 mg twice daily or 2.5 mg twice daily) or placebo and treated for 16 weeks. The results showed that only the high dose of 10 mg twice daily improves lean and fat mass after 16 weeks of treatment. Results of the functional data showed that only handgrip strength significantly increased after 16 weeks in the low‐dose group and in the high‐dose group of treatment but stair‐climbing power and 6 min walk distance were left unaffected. This study gives rise to the question whether or not beta‐blockers can be viewed as having a class effect in the treatment of cancer cachexia.

Loss of skeletal muscle mass and strength plays a significant pathological role in the progression of a wide variety of disorders associated with ageing and catabolic conditions. Interestingly this year, the frailty studies FLAGSHIP and SPRINT‐T were presented. These big data cohort studies are recruiting patients, and we are waiting for the first results maybe in the next meeting.

## Conclusions

From basic science, new therapeutic targets were shown including the TWEAK–Fn14–NF‐*κ*B–MuRF1–myosin heavy chain protein degradation cascade and the neutralization of tumour‐derived parathyroid‐hormone‐related protein, as well as the influence and the role of the gut microbiota in the therapeutic management of cancer and associated cachexia. Effective treatments were REGN1033, ghrelin, and ghrelin receptor agonist anamorelin and enobosarm.

## Conflict of interest

None declared.

## References

[1] Ebner N , Steinbeck L , Doehner W , Anker SD , von Haehling S . Highlights from the 7th Cachexia Conference: muscle wasting pathophysiological detection and novel treatment strategies. J Cachexia Sarcopenia Muscle 2014;5:27–34. 24595460 10.1007/s13539-014-0136-z PMC3953317

[2] Ebner N , Werner CG , Doehner W , Anker SD , von Haehling S . Recent developments in the treatment of cachexia: highlights from the 6th Cachexia Conference. J Cachexia Sarcopenia Muscle 2012;3:45–50. 22460618 10.1007/s13539-012-0061-y PMC3302986

[3] Ebner N , Springer J , Kalantar‐Zadeh K , Lainscak M , Doehner W , Anker SD , et al. Mechanism and novel therapeutic approaches to wasting in chronic disease. Maturitas 2013;75:199–206. 23664695 10.1016/j.maturitas.2013.03.014

[4] Polge C , Claustre A , Deval C , Béchet D , Combaret L , Attaix D , et al. Identification of E2 enzymes involved in MuRF1‐dependent skeletal muscle atrophy. J Cachexia Sarcopenia Muscle 2016;7:627, Abstract 1‐03.

[5] Kneppers AAEM , Pansters NAM , Leermakers PA , Verdijk LB , van Loon LJC , Schols AMWJ , et al. Parallel activation of autophagy and myogenic signalling during muscle mass recovery following disuse‐atrophy. J Cachexia Sarcopenia Muscle 2016;7:628, Abstract 1‐05.

[6] Musolino V , Bosco F , Nucera S , Giancotti LA , Ilari S , Lauro F , et al. New insights into muscle atrophy: role of autophagy in a model of chronic sciatic nerve constriction. J Cachexia Sarcopenia Muscle 2016;7:629, Abstract 1‐08.

[7] Talbert EE , Yang J , Mace TA , Farren MR , Farris AB , Young GS , et al. Dual inhibition of MEK and PI3K/Akt rescues cancer cachexia through both tumor extrinsic and intrinsic activities. Mol Cancer Ther 2016;3: pii: molcanther.0337.2016. 10.1158/1535-7163.MCT-16-0337 PMC5292063 27811010

[8] Lodka D , Pahuja A , Geers‐Knörr C , Scheibe RJ , Nowak M , Hamati J , et al. Muscle RING‐finger 2 and 3 maintain striated‐muscle structure and function. J Cachexia Sarcopenia Muscle 2016;7:165–180. 27493870 10.1002/jcsm.12057 PMC4863828

[9] Kowalski K , Archacki R , Archacka K , Stremińska W , Paciorek A , Gołąbek M , et al. Stromal derived factor‐1 and granulocyte‐colony stimulating factor treatment improves regeneration of Pax7‐/‐ mice skeletal muscles. J Cachexia Sarcopenia Muscle 2016;7:483–496. 27239402 10.1002/jcsm.12092 PMC4863826

[10] Marino FE , Risbridger G , Gold E . Activin‐βC modulates cachexia by repressing the ubiquitin‐proteasome and autophagic degradation pathways. J Cachexia Sarcopenia Muscle 2015;6:365–380. 26673867 10.1002/jcsm.12031 PMC4670746

[11] Ebner N , Springer J , Kalantar‐Zadeh K , Lainscak M , Doehner W , Anker SD , et al. Mechanism and novel therapeutic approaches to wasting in chronic disease. Maturitas 2013;75:199–206. 23664695 10.1016/j.maturitas.2013.03.014

[12] Garagarza C , Flores AL , Valente A . Correlation between handgrip strength and muscle mass with biochemical and body composition parameters. J Cachexia Sarcopenia Muscle 2017;8:167, Abstract 1‐54.

[13] Szulc P , Chapurlat R . Accelerated grip strength decline in older men with poor health and hormonal deficits—the prospective STRAMBO study. J Cachexia Sarcopenia Muscle 2016;7:631, Abstract 1‐13. 10.1002/jcsm.12066 PMC4864191 27239407

[14] Stimpson SA , Leonard MS , Clifton LG , Poole JC , Turner SM , Shearer TW , et al. Longitudinal changes in total body creatine pool size and skeletal muscle mass using the D3‐creatine dilution method. J Cachexia Sarcopenia Muscle 2013;3:217–223. 10.1007/s13539-013-0110-1 PMC3774916 23797207

[15] Ebner N , von Haehling S . Unlocking the wasting enigma: highlights from the 8th Cachexia Conference. J Cachexia Sarcopenia Muscle 2016;7:90–94. 27128291 10.1002/jcsm.12106 PMC4799863

[16] Li J , Frederics M , Suragani RNVS , Pearsall SR , Kumar R . ACE‐2494, a systemically acting transforming growth factor‐β superfamily ligand trap, prevents and restores muscle loss and weakness in disuse atrophy mice. J Cachexia Sarcopenia Muscle 2017;8:168, Abstract 1‐56.

[17] Ábrigo J , Márquez‐Miranda V , Rivera JC , Araya‐Durán I , Aravena J , Pacheco N , et al. New formulation based on anti‐atrophic peptides and dendrimers for skeletal muscle atrophy treatment. J Cachexia Sarcopenia Muscle 2016;7:626, Abstract 1‐01.

[18] Rivera JC , Abrigo J , Chiong M , Bader M , Santos RA , Brandan E , et al. Endotoxin‐induced autophagy dependent on Beclin1/Bcl2 complex is decreased by angiotensin‐(1‐7) in skeletal muscle. J Cachexia Sarcopenia Muscle 2016;7:626, Abstract 1‐02.

[19] Paddon‐Jones D , Rasmussen BB . Dietary protein recommendations and the prevention of sarcopenia. Curr Opin Clin Nutr Metab Care 2009;12:86–90. 19057193 10.1097/MCO.0b013e32831cef8b PMC2760315

[20] Calder PC , Laviano A , Lonnqvist F , Muscaritoli M , Öhlander M , Schols A . Targeted medical nutrition for cachexia in chronic obstructive pulmonary disease (COPD): a randomized, double‐blind controlled trial. J Cachexia Sarcopenia Muscle 2017;7:174, Abstract 2‐15. 10.1002/jcsm.12228 PMC5803606 28891198

[21] Karaa A , Cohen BH , Goldstein A , Vockley J , Haas R . MMPOWER: the effect of treatment with elamipretide in patients with genetically confirmed primary mitochondrial disease. J Cachexia Sarcopenia Muscle 2017;7:170, Abstract 1‐61.

[22] Coats AJS , Ho GF , Prabhash K , von Haehling S , Tilson J , Brown R , et al. For and on behalf of the ACT‐ONE study group. Espindolol for the treatment and prevention of cachexia in patients with stage III/IV non‐small cell lung cancer or colorectal cancer: a randomized, double blind, placebo‐controlled, international multicentre phase II study (the ACT‐ONE trial). J Cachexia Sarcopenia Muscle 2016;7:355–365. 27386169 10.1002/jcsm.12126 PMC4929828

[23] von Haehling S , Morley JE , Coats AJS , Anker SD . Ethical guidelines for publishing in the Journal of Cachexia, Sarcopenia and Muscle: update 2015. J Cachexia Sarcopenia Muscle 2015;6:315–316. 26672494 10.1002/jcsm.12089 PMC4670739

